# Transgenerational effects of ungulates and pre-dispersal seed predators on offspring success and resistance to herbivory

**DOI:** 10.1371/journal.pone.0207553

**Published:** 2018-12-12

**Authors:** Martin Aguirrebengoa, Maite García-Planas, Caroline Müller, Adela González-Megías

**Affiliations:** 1 Departamento de Zoología, Universidad de Granada, Granada, Spain; 2 Department of Chemical Ecology, Bielefeld University, Bielefeld, Germany; Helmholtz Zentrum Munchen Deutsches Forschungszentrum fur Umwelt und Gesundheit, GERMANY

## Abstract

Herbivorous mammals and insect pre-dispersal seed predators are two types of herbivores that, despite their functional and morphological differences, tend to severely impact many plant species, highly decreasing their seed production and even imperiling the performance of their offspring through transgenerational effects. However, how they influence offspring resistance to herbivory remains largely unknown. In this study we experimentally examined the effects of ungulates and pre-dispersal seed predators on seed quality as well as on the emergence, survival and resistance to herbivory of the seedlings of a semiarid herb. We found that ungulates reduced seedling recruitment but increased seedling resistance to leaf miners. These effects were probably a consequence of insufficient carbon provisioning in seeds that reduced seed viability and provoked carbon limitation in seedlings. Pre-dispersal seed predators did not influence seedling recruitment, but seedlings from mothers damaged by ungulates and by pre-dispersal seed predators suffered less herbivory by grasshoppers. Remarkably, intra-individual differences in damage by pre-dispersal seed predators affected the rate of damage underwent by seedlings. That is, seedlings derived from fruits attacked by seed predators were more resistant to herbivores than siblings derived from un-attacked fruits in plant populations exposed to ungulates. To our knowledge, this is the first study reporting variation in transgenerational-induced resistance of seedlings from the same maternal plant. This study is a valuable contribution to the understanding of transgenerational effects of multiple herbivores and their implications for a deeper comprehension of the natural systems in which they co-occur.

## Introduction

Plants are subjected to varying stresses during most of their life mainly because they are unable to escape by moving to another place. However, they have evolved multiple phenotypic traits enabling them to handle stressful conditions [1]. It is well known that not only parental genotypes but also the parental environment determine the expression of traits in plants [2,3]. When both parental and offspring environments are alike they may attenuate any detrimental effect on fitness caused by the stressful conditions [2,3]. There is an intense debate trying to elucidate whether changes in the offspring phenotype are merely a response to the allocation of the resources made by the parents (maternal provisioning) or whether they are adaptive transgenerational effects [3–5]. Regardless of the mechanisms, the parental environment can potentially affect not only plant population dynamics but also evolutionary processes [3].

Transgenerational responses to the parental environment associated with abiotic stresses (e.g. nutrients, light, temperature) have been recognized for many years [2]. More recently, the role of biotic stresses in altering offspring phenotype has also been highlighted [3,6–9]. In particular, several studies have shown that herbivores can alter offspring success through transgenerational effects [6,10–12]. Herbivore-mediated transgenerational effects can alter offspring phenotypes by affecting traits associated with offspring performance (e.g. height, biomass) or defense (e.g. trichome density, chemical defense) [8–14]. These biotic-mediated transgenerational effects can help offspring to better cope with herbivores, and consequently increase its fitness [6,10–12]. Therefore, the quantification of transgenerational effects of herbivory may provide a better understanding of the complex impact of herbivores on plant fitness [15].

During their lifespan plants are usually subjected to the attack of numerous herbivores. Herbivore effects on plants vary according to herbivore characteristics such as their specialization degree, size, or the part of the plant consumed [16–18]. Large herbivores such as mammals are important disturbance agents in most terrestrial ecosystems both by their direct and indirect impacts on plants [19]. They alter nutrient cycling, modulate succession and vegetation dynamics and can generate spatial heterogeneity [20,21]. Herbivorous mammals impact plants mostly by trampling and/or consuming them [19]. These impacts entail a significant decrease in the number of seeds produced by damaged plants. Additionally, mammalian herbivores may diminish the quality of the seeds produced by the attacked plants, greatly lessening their germination, emergence and establishment [22–25]. Seed quality is related to the resource investment into the seeds by the maternal plant, and for many species it is critical for germination and seedling survival [6,7,10,26,27].

Herbivore mammals also have important indirect effects on plants by consuming herbivorous insects associated with the plant [28–30] or by disrupting the interaction between plants and their mutualists [31,32]. The interaction between herbivore mammals and phytophagous insects is generally asymmetric, due to their huge differences in size [28,33]. Herbivore mammals especially affect endophytic insects, including pre-dispersal seed predators (PSPs) [29,34]. PSPs are important components of ecological communities and usually have negative effects on plants by reducing seed production, seed germination and seedling establishment [35–37]. However, there are exceptions with some pre-dispersal seed predators having neutral effects (see [37]) or even increasing host plant seed production [Aguirrebengoa et al. under review]. Interestingly, plants often exhibit within-individual phenotypic variation in organs such as reproductive tissues, with seeds being one of the most variable structures [38,39]. This can have important implications for the interactions between plants and PSPs. In fact, the action of PSPs may alter resource allocation processes within the plant and promote a local induction of defenses, and therefore enhance within-plant variability in seed attributes and entail significant transgenerational consequences.

Although there are some studies examining the effects of mammals and PSPs on seed quality and their consequences on seed germination and seedling survival, none than we are aware of go a bit further and examine the effect of both herbivores on offspring resistance to herbivory. In *Moricandia moricandioides*, a predominantly semelparous Brassicaceae herb inhabiting dryland environments in the Iberian Peninsula, PSPs induce a compensatory response by enhancing plant height and flower production which results in overcompensation in seed number [Aguirrebengoa et al. under review]. In a 7-year study, we found that ungulates negatively affected *M*. *moricandioides* populations by reducing plant density and increasing the variability in population size among years. Similarly, ungulates decreased *M*. *moricandioides* fitness by reducing PSPs attack rate [González-Megías et al. in prep.]. In the present study, we examined the effects of long-term ungulate pressure and of PSPs on seed quality and seedling emergence and survival. We hypothesized negative effects of both herbivores in line with a previous study on *M*. *moricandioides* that revealed that seeds derived from plants undergoing root and floral herbivory were of lower quality and performed worse during early establishment [40]. We then determined whether the exposure to both herbivore types by parental plants increased the resistance of the offspring to the damage by insect herbivores. We predicted distinct outcomes in response to both herbivore types due to their specialization degree (generalist *versus* specialist), size (the magnitude of damage and the part of the plant consumed), and net effect on the plant (negative for ungulates and positive by PSPs). Finally, because phenotypic difference can be found even between individual parts of single plant individual, we determined whether maternally-mediated effects occurred within plants by comparing the fate of seeds and siblings from fruit with and without PSPs, and whether these differences are influenced by the exposure of maternal plants to ungulates.

## Materials and methods

### Study area

The experimental study area is located at the Barranco del Espartal, a seasonal watercourse in the arid Guadix-Baza Basin (Granada Province, southeastern Spain). The climate at the study area is continental Mediterranean with strong temperature fluctuations (ranging from -14°C to up to 45°C) and high seasonality (hot summers, cold winters). Annual precipitation rarely exceeds 300 mm and potential evapotranspiration is 3–4 times higher than precipitation, severely conditioning vegetation diversity and cover [41]. The soil is characterized by a sandy–loam texture, high pH, low water-retention capacity and high salinity. The vegetation is an arid open shrub-steppe dominated by *Artemisia herba-alba* Asso, *A*. *barrelieri* Bess. (Asteraceae), *Salsola oppositifolia* Desf. (Amaranthaceae), *Stipa tenacissima* L. (Poaceae), *Retama sphaerocarpa* L. (Fabaceae), *Ononis tridentata* L. (Fabaceae) and *Lygeum spartum* L. (Poaceae) [41].

The study area comprises private and governmental land and suffered different human pressure: some of the properties are used exclusively for hunting (mainly rabbits, hares and partridges) and grazing by domestic ungulates has not been permitted for at least the last 25 years. The rest of the area is open to be used by domestic ungulates (mainly sheep) and numbers has varied from 500 to 700 sheep (50 or more years ago) to around 100 sheep (since 2011 to today). In the areas to which they have access, ungulates cause a pronounced impact on annual and short-lived plant populations [42].

### Model system

The short-lived Brassicaceae species *Moricandia moricandioides* (Boiss.) Heywood is highly abundant in the study area and was used as a model system. *Moricandia moricandioides* plants grow as a vegetative rosette during winter, and produce reproductive stalks during late spring [42]. Most individuals reproduce only once, but few individuals are able to resprout the next season and reproduce more than one year (less than 10% of the population). As a Brassicales, *M*. *moricandioides* is provided with potent chemical defenses, with the glucosinolate-myrosinase system being probably the most important defense [43]. In particular, seeds are expected to be one of the most well defended tissues, due to their value in terms of fitness [44].

The pre-dispersal seed predator *Crossobela trinotella* (Herrich-Schaffer, 1856; Lepidoptera, Gelechiidae) is among the most abundant herbivores of *M*. *moricandioides*. The attack rate of *C*. *trinotella* fluctuates between years and among populations [González-Megías et al. in prep.]. This Brassicaceae specialist oviposits on flowers or immature fruits, and the caterpillar develops inside the fruits feeding on seeds [45]. There can be more than one caterpillar per fruit, each of them eating 8–12 seeds (fruits have usually 20–60 seeds).

### Experimental set-up and data collection

We selected 12 populations of *M*. *moricandioides* in the study area (S1 Fig); six populations located in areas with ungulate presence (UNG+), and six populations in areas inaccessible for ungulates (UNG-). As populations, we refer to patches of *M*. *moricandioides* isolated from other *M*. *moricandioides* patches or individuals for at least 100 m.

During July-August 2013, we selected 15 random plants from each of the 12 experimental populations. We counted the total number of fruits per plant in the field and collected five fruits of each of the 15 plants per population (900 fruits in total). Once in the lab and during autumn, we recorded the presence (PSP+) or absence (PSP-) of the pre-dispersal seed predator at both plant and fruit level (see S2 Fig flow diagram). The percentage of fruits with seed predators found during the study year was 13% for the surveyed populations.

*C*. *trinotella* was the main seed predator found in the fruits (84% of the PSPs), followed by an unidentified curculionid. Only in three fruits (0.33% of fruits) both PSP species were present together. Due to the predominance of *C*. *trinotella*, seed predator identity was not considered in the following analyses.

### Seed quality

Seed quality was measured as seed mass, C and N contents and the concentrations of chemical defenses (glucosinolates) in the seed. Each of these attributes was estimated in a subset of plants (S2 Fig). We weighed groups of 10 seeds from each fruit to estimate the mean weight of a seed for each fruit (with a 0.01 mg accuracy Sartorius Cubis MSE-125P precision balance). Mean seed weight from 209 fruits from 62 plants that belonged to populations with and without ungulates, and plants with and without PSPs was estimated. Fruit sample size across treatments varied between plants (see sample sizes in S2 Fig) and at within-plants (fruits with and without PSPs from the same plant; S2 Fig).

Carbon and nitrogen contents in seeds were measured at fruit level for another set of seeds in five UNG+ and five UNG- populations of *M*. *moricandioides*. A pool of ~15 seeds from each of 197 fruits from 64 plants were analyzed (range 1–5 fruits per plant), most of the plants being the same than those used for assessing seedling emergence (see below). Fruit selection was done to ensure a minimum of 10 fruits per treatment (see between-plant and within-plant sample sizes in S2 Fig). Carbon and nitrogen contents in seeds were measured using a CHN Elemental Analyzer (Thermo Scientific Flash 2000) and the C/N ratio calculated.

Glucosinolate concentrations were quantified for another a set of seeds from five UNG+ and six UNG- populations of *M*. *moricandioides*. For glucosinolate concentrations at between-plant level, a pool of 20–25 seeds from all collected fruits of 99 plants was analyzed, many of the plants being the same than those used for assessing seedling emergence rate, and with a varying sample size across treatments (S2 Fig). For glucosinolate concentrations in fruits within the same plant, a pool of 20–25 seeds from all fruits without PSPs was compared to a pool of 20–25 seeds from all fruits with PSPs from the same plant. Due to limitations in seed number, this analysis was restricted to 19 plants from 6 UNG- populations. Seeds were ground and extracted three times in 80% methanol, adding *p*-hydroxybenzyl glucosinolate (sinalbin) as an internal standard at the first extraction. Glucosinolate extraction and conversion to desulfoglucosinolates as well as measurement by high performance liquid chromatography coupled to a diode-array detector (1200 Series, Agilent Technologies) were done as previously described [46]. Desulfoglucosinolates were identified by comparison of UV-spectra and retention times to those identified in earlier studies [46]. Peaks were integrated at 229 nm and response factors of 1 for aliphatic glucosinolates and 0.26 for indolic glucosinolates were considered and related to the internal standard (response factor 0.5) and sample dry weight for calculation of concentrations.

### Seedling emergence

To determine the effects of ungulates and PSPs on seedling emergence rate, ten *M*. *moricandioides* populations were selected (five UNG+ and five UNG-). In each population, up to eight plants were selected according to the presence of PSPs (four PSP+ and four PSP-). For each plant, we selected up to 25 intact seeds (range 8–25) from each of the five fruits collected from the field (S2 Fig). A total of 6317 seeds were planted in black peat moss between 10 and 12-Dec-2013, in 34 seedbeds at a greenhouse with natural temperature and photoperiod conditions. Seedbeds were rotated in the same direction every other day to avoid possible location effects.

Seedling emergence was supervised every other day from the planting day until the end of January 2014 (last emergence was on 24-Jan-2014), when emergence of *M*. *moricandioides* stopped. Additionally, we calculated emergence time as the time lapse from planted to emerge.

### Transgenerational effects of ungulates and pre-dispersal seed predators on seedlings: Field experiments

We carried out two experiments in the field to test transgenerational effects on seedling survival, performance and herbivory on *M*. *moricandioides*:

#### Experiment 1: Between-plant level

For this experiment, we selected seedlings belonging to six populations (three UNG+ and three UNG-). Ten seedlings from 6 mother plants (three PSP+ and three PSP-) per population were randomly positioned in 5 blocks in the study area (72 seedlings x block: 360 seedlings; S2 Fig).

#### Experiment 2: Within-plant level

Following the same methodology as in experiment 1, we selected seedlings belonging to mother plants with PSPs from 8 populations (four UNG+ and four UNG-). From each plant (6 plants from UNG+ and 5 from UNG- populations), we selected 10 seedlings derived from fruits with PSPs and other 10 seedlings derived from fruits without PSPs. A total of 220 seedlings were randomly positioned in each of 5 designed blocks (44 seedlings x block; S2 Fig).

Seedlings for both experiments were planted in the field between 12 and 14-Feb-2014. All selected seedlings had two young leaves when planted. During the experiment (from 17-Feb-2014 to 27-Apr-2014), the number of leaves attacked by herbivores, the identity of the herbivore, and plant survival were noted three times per week. The number of leaves per plant was counted once a week. Herbivory on seedlings corresponded almost exclusively to three herbivorous insect guilds, which were differentiated due to their distinguishable damage on leaves: generalist chewers (predominantly acridid grasshoppers, Acrididae, Orthoptera), specialist chewers (*Phyllotreta spp*., Chrysomelidae, Coleoptera) and leaf miners (Diptera). Herbivory was analyzed as the total number of leaves attacked by all herbivores, and by each individual guild. The number of attacked leaves was monitored rather than the amount of leaf consumed because leaves were too small, and because grasshopper and chrysomelid attacks on leaves many times implied the total or almost total consumption of leaves.

### Statistical analyses

#### A1) Effects of ungulates and pre-dispersal seed predators on seed quality and seedling emergence rate: Between-plant level

We analyzed the effects of both herbivore types (ungulates and PSPs) and their interaction on seed traits and seedling emergence. Seed quality traits (mass, carbon and nitrogen content, C/N ratio, and aliphatic and indolic glucosinolate concentrations) were analyzed using linear mixed models (LMMs) with REML-based estimations. Glucosinolate concentrations were log (x+1) transformed. Because both maternal lineage and population origin could influence the traits of interest, several models with different random structures were tested for all these variables to control for origin (S1 Appendix). Plant identity was always included as random factor, with the exception of glucosinolate concentrations because we had a single measure per plant.

Seedling emergence rate and time were analyzed using generalized linear mixed models (GLMMs), emergence rate with a binomial distribution with logit link function and emergence time with Poisson distribution with log link function. Several models with different random structures were also tested for these variables (S1 Appendix).

#### A2) Effects of ungulates and pre-dispersal seed predators on seed quality and seedling emergence rate: Within-plant level

Within-plant level effects on seed traits and seedling emergence were estimated from the subsample of plants with PSPs from both UNG+ and UNG- populations, comparing fruits with and without PSPs from the same plants. Ungulate presence/absence was included as a factor in all analyses to determine whether ungulates affect the potential within-plant effect of PSPs. We were interested in the single effect of PSPs and the interactive effect of the two types of herbivores rather than in the single effect of ungulates (which cannot be tested within the same plant). However, to avoid confounding the readers, when the single effect of ungulates was significant in our analysis, it is indicated in the result section. Several models with different random structures were also tested for these variables with fruit nested within plant (S1 Appendix).

#### B1) Transgenerational effects of ungulates and pre-dispersal seed predators on seedlings: Between-plant level

LMMs and GLMMs were also used to determine the effects of ungulates, PSPs and their interaction on variables from the between-plant level field experiment (Field Experiment 1). Maternal plant identity was always included as random factor to control for origin, and block was tested for all variables as a random factor and included when it contributed to model improvement (S1 Appendix). Seedling leaf production was analyzed with Gaussian distribution, and seedling survival rate with a binomial distribution. In the case of herbivore-related variables, zero-truncated Poisson and zero-inflated Poisson models with log link function were performed (S1 Appendix).

#### B2) Transgenerational effects of ungulates and pre-dispersal seed predators on seedlings: Within-plant level

Within-plant level effects in offspring were estimated from the selected mother plants with PSPs from both UNG+ and UNG- populations for the within-plant level field experiment (Field Experiment 2), comparing siblings from fruits with and without PSPs. Maternal ungulate presence/absence was included as factor, maternal plant identity was included as random factor, fruit of origin was nested within plant and block was tested as a random factor for all variables (S1 Appendix). Seedling leaf production, seedling survival rate and herbivore-related variables were analyzed with the same distributions described for between-plant level effects.

All models for each variable from the above sections were compared by three Information Criteria (IC), Akaike’s Information Criterion (AIC), small sample size corrected Akaike’s Information Criterion (AICc) and Bayesian Information Criterion (BIC), and weighed when there were >2 models (S1 Appendix). ICs values were used for model selection on variables with 2 possible models, in which strictly best models (lowest IC values) were chosen. On variables for which there were >2 possible models, model weighing showed relatively high support for choosing the best model based on lowest IC values. Results were very similar for the three IC values and weights, and on the few cases in which there was divergence between them, BIC was used for model selection, as it tends to favor more parsimonious models [47]. All analyses were performed in R 3.1.1 [48] using *nlme* [49] and *lme4* [50] packages, and with the *glmmADMB* [51] package in the case of zero-truncated and zero-inflated models.

## Results

### A) Effects of herbivores on seed quality and seedling emergence rate

**Fig 1:**
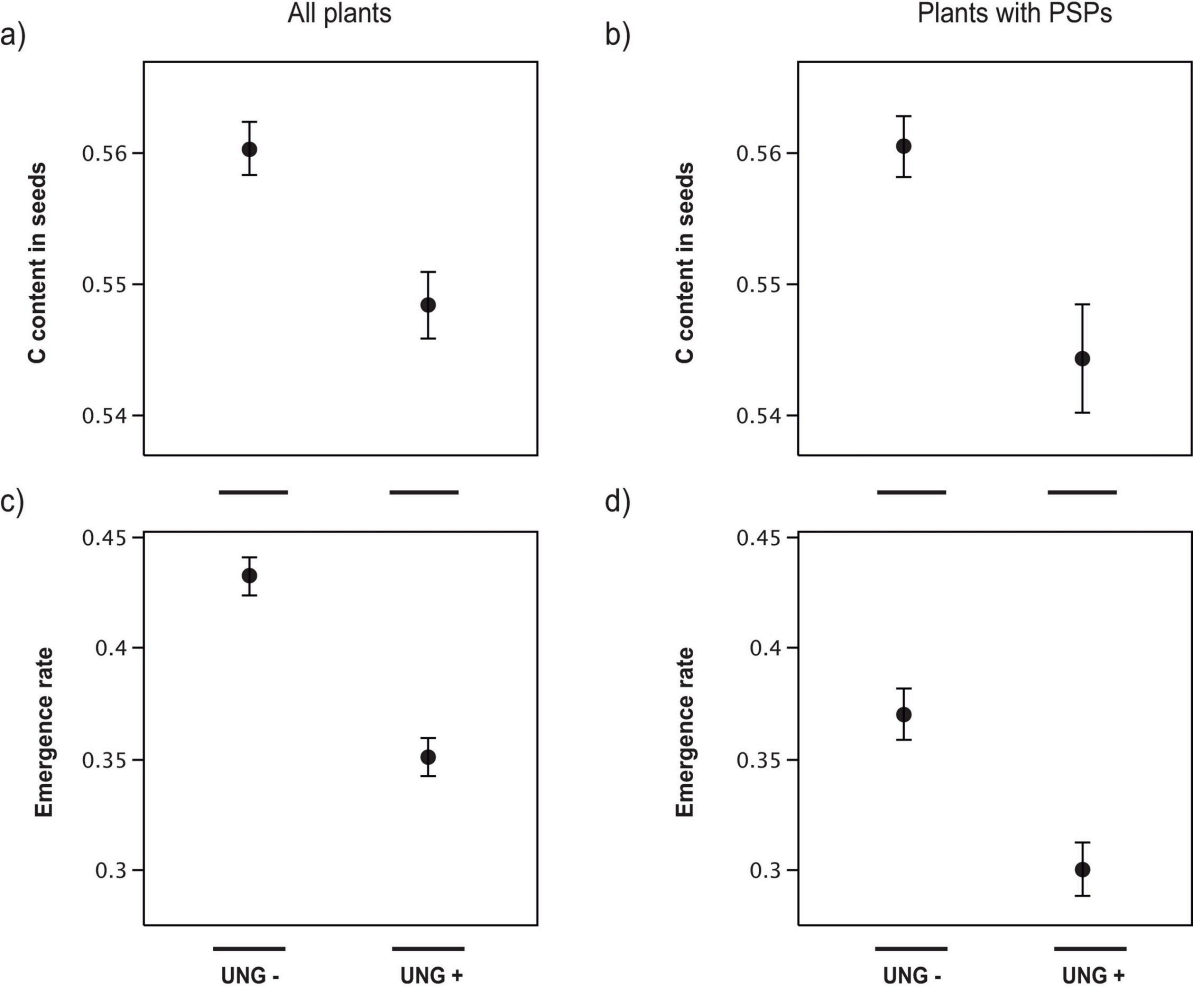
Effects of ungulates on seed carbon content and seedling emergence rate. Ungulate effect on seed carbon content of *Moricandia moricandioides* at between-plant (a) and at within-plant level (b). Ungulate effect on emergence rate at between-plant (c) and at within-plant level (d). Mean ± SE.

There was no effect of herbivores on seed mass at neither between-plant level nor within-plant level (Table 1). Carbon content in seeds was negatively affected by ungulates at between-plant level and within-plant level (Fig 1; Table 1). There was no effect of herbivores on nitrogen content and C/N ratio at between-plant level or at within-plant level (Table 1).

**Table 1:** Ungulate and PSP effects on seed quality and glucosinolate concentrations at between-plant and within-plant levels of *Moricandia moricandioides*.

	Ungulate			PSP			Ungulate x PSP		
	*F*	*P*	DF	*F*	*P*	DF	*F*	*P*	DF
**Between-plant level**									
*Seed quality*									
Seed mass	0.00	0.97	1, 58	0.49	0.48	1, 58	1.92	0.17	1, 58
Carbon in seeds	**4.90**	**0.031**	1, 53	0.20	0.65	1, 53	2.20	0.14	1, 53
Nitrogen in seeds	0.00	0.97	1, 8	0.11	0.74	1, 45	1.00	0.32	1, 45
C/N ratio in seeds	0.26	0.62	1, 8	0.23	0.63	1, 45	0.27	0.60	1, 45
*Glucosinolates*									
Aliphatic glucosinolates in seeds	0.00	0.97	1, 95	0.45	0.50	1, 95	0.39	0.53	1, 95
Indolic glucosinolates in seeds	2.16	0.14	1, 95	0.20	0.65	1, 95	1.89	0.17	1, 95
**Within-plant level**									
*Seed quality*									
Seed mass	0.86	0.36	1, 28	0.04	0.84	1, 17	0.25	0.62	1, 17
Carbon in seeds	**8.23**	**0.007**	1, 27	0.30	0.59	1, 14	0.00	0.97	1, 14
Nitrogen in seeds	0.33	0.57	1, 27	0.31	0.58	1, 14	1.40	0.25	1, 14
C/N ratio in seeds	0.09	0.76	1, 27	0.15	0.70	1, 14	1.43	0.25	1, 14
*Glucosinolates*									
Aliphatic glucosinolates in seeds	-	-		0.23	0.63	1, 6	-	-	
Indolic glucosinolates in seeds	-	-		0.16	0.69	1, 6	-	-	

Significant effects (*P* < 0.05) are shown in bold.

There was no effect of ungulates or PSPs on the concentration of total glucosinolates, aliphatic glucosinolates, or indolic glucosinolates in seeds at between-plant level or within-plant level (Table 1).

Ungulates negatively affected emergence rate at between-plant level (Fig 1C) and within-plant level (Fig 1D; Table 2). Emergence time was not affected by ungulates or PSPs at any level (Table 2).

**Table 2:** Ungulate and PSP effects on seedling emergence and emergence time at between-plant and within-plant levels of *Moricandia moricandioides*.

	Ungulate			PSP			Ungulate x PSP		
	*χ*^*2*^	*P*	DF	*χ*^*2*^	*P*	DF	*χ*^*2*^	*P*	DF
**Between-plant level**									
Emergence rate	**9.85**	**0.001**	1, 57	2.90	0.08	1, 57	0.97	0.32	1, 57
Emergence time	0.62	0.43	1, 56	0.14	0.70	1, 56	0.02	0.88	1, 56
**Within-plant level**									
Emergence rate	**4.59**	**0.032**	1, 32	0.18	0.67	1, 30	1.58	0.20	1, 30
Emergence time	0.04	0.84	1, 31	0.45	0.51	1, 28	2.44	0.13	1, 28

Significant effects (*P* < 0.05) are shown in bold.

### B) Transgenerational effects of ungulates and pre-dispersal seed predators on seedlings

#### Experiment 1: Between-plant level

There was no transgenerational effect of ungulates or PSPs on the number of leaves produced per seedling or on seedling survival rate (Table 3).

**Fig 2:**
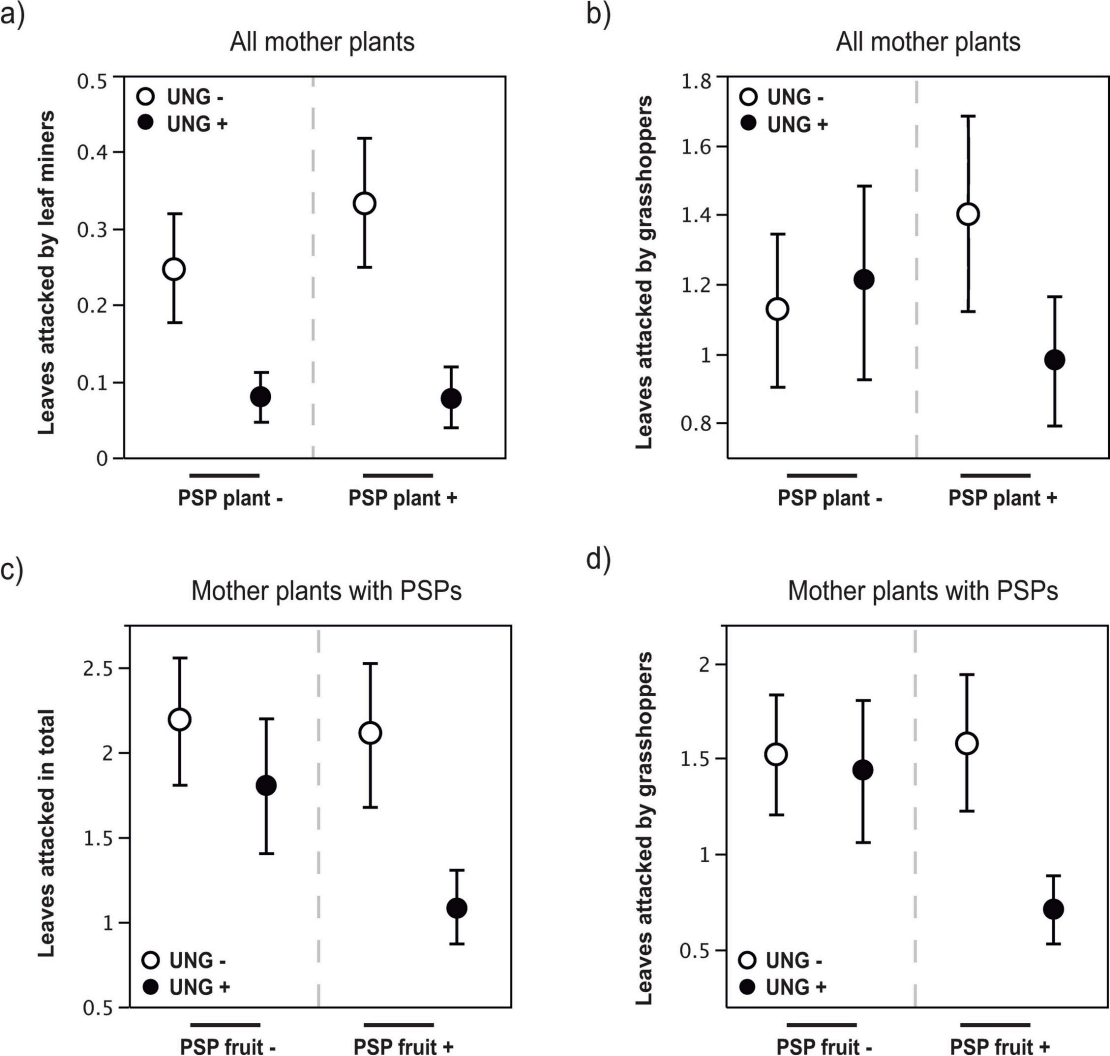
Transgenerational effects of ungulates and PSPs on next generation seedling herbivory. a) Transgenerational ungulate and PSP effects on attacked leaves of *Moricandia moricandioides* seedlings by leaf miners at between-plant level (*Field Experiment 1*). b) Transgenerational ungulate and PSP effects on total number of attacked leaves in seedlings at between-plant level (*Field Experiment 1*). c) Transgenerational ungulate and PSP effects on total number of attacked leaves in seedlings at within-plant level (*Field Experiment 2*). d) Transgenerational ungulate and PSP effects on total number of attacked leaves in seedlings by grasshoppers at within-plant level (*Field Experiment 2*). Mean ± SE.

There was no effect of previous generation herbivores on the number of leaves attacked in total or by chrysomelid beetles (Table 3). However, there was a negative transgenerational effect of ungulates on the number of leaves attacked by leaf miners (Fig 2, Table 3). The number of leaves attacked by grasshoppers was lower in seedlings derived from maternal plants that suffered from both herbivores, as shown by the significant interaction term (Fig 2B, Table 3).

**Table 3:** Ungulate and PSP effects on seedling performance and herbivory (seedling resistance) at between-plant and within-plant levels of *Moricandia moricandioides*.

	Ungulate			PSP			Ungulate x PSP		
	*χ*^*2*^ */ F*	*P*	DF	*χ*^*2*^ */ F*	*P*	DF	*χ*^*2*^ */ F*	*P*	DF
**Between-plant level**									
*Seedling performance*									
Survival rate	0.00	0.97	1, 32	0.00	0.97	1, 32	0.42	0.51	1, 32
Produced leaves	0.67	0.41	1, 32	0.34	0.56	1, 32	1.25	0.27	1, 32
*Seedling resistance*									
Leaves attacked by chrysomelids	0.73	0.39	1, 32	2.20	0.13	1, 32	0.03	0.86	1, 32
Leaves attacked by leaf miners	**4.61**	**0.031**	1, 32	0.81	0.36	1, 32	0.15	0.69	1, 32
Leaves attacked by grasshoppers	0.00	0.97	1, 32	1.41	0.23	1, 32	**6.19**	**0.012**	1, 32
Leaves attacked in total	0.65	0.42	1, 32	0.05	0.82	1, 32	0.27	0.60	1, 32
**Within-plant level**									
*Seedling performance*									
Survival rate	**3.98**	**0.046**	1, 9	0.07	0.79	1, 9	0.10	0.75	1, 9
Produced leaves	2.12	0.20	1, 9	0.04	0.84	1, 9	0.00	0.97	1, 9
*Seedling resistance*									
Leaves attacked by chrysomelids	0.22	0.63	1, 9	0.65	0.42	1, 9	2.03	0.15	1, 9
Leaves attacked by leaf miners	2.06	0.15	1, 9	0.58	0.44	1, 9	1.05	0.30	1, 9
Leaves attacked by grasshoppers	0.03	0.86	1, 9	0.03	0.86	1, 9	3.51	0.06	1, 9
Leaves attacked in total	1.65	0.19	1, 9	2.66	0.10	1, 9	**5.86**	**0.015**	1, 9

*F* values are shown for the variable Produced leaves, *χ*^*2*^ values are shown for the rest. Significant effects (*P* < 0.05) are shown in bold.

#### Experiment 2: Within-plant level

There was no transgenerational effect of herbivores on the number of leaves produced per seedling (Table 3), but there was a negative transgenerational effect of ungulates on seedling survival rate in the field (UNG- = 0.64 ± 0.05, UNG+ = 0.45 ± 0.06; Table 3).

There was an interactive transgenerational effect of ungulates and PSPs on seedling herbivores (Table 3). The total number of leaves attacked by herbivores was lower in seedlings derived from maternal plants under both ungulates and PSPs exposure (Fig 2C). A similar but marginally significant transgenerational effect was found for the number of leaves attacked by grasshoppers (Fig 2D, Table 3). There was no effect of previous generation herbivores on the number of leaves attacked by chrysomelid beetles or leaf miners (Table 3).

## Discussion

### Effects of herbivores on seed quality and seedling emergence rate

The present study supports the idea that the maternal biotic environment has important consequences for the offspring. In particular, ungulates had strong and significant negative effects on *M*. *moricandioides* offspring. Ungulates effects on the mother plants significantly reduced the emergence rate of their seedlings in greenhouse conditions. This transgenerational effect of ungulates on emergence rate has been previously reported for other herbs and shrubs [22,52]. We presume that this effect may even be underestimated, as any effects of herbivores on seedling emergence are expected to be much higher under field conditions [40]. Furthermore, damage by ungulates to mother plants also significantly lessened the survival of seedlings in field conditions. Interestingly, this effect was mostly evident in plants attacked also by PSPs, suggesting that PSPs may also impinge some, albeit weak, transgenerational effects. Therefore, our results suggest that ungulates may have much larger long-term effects on *M*. *moricandioides* population than inferred from their solely effects on seed production. The current study, thus, indicates that an accurate and precise estimate of ungulate effects on plant population dynamics requires not just quantifying their intra-generational impacts but also the transgenerational consequences of the damage. When these two processes act synergistically, the long-term negative effects on plant populations are greatly amplified and cannot be deduced from studies focusing exclusively on intra-generational effects.

Many theoretical and empirical studies have found that the effects of herbivores on plant performance are stronger in short-lived herbs than in perennials [17,53]. This is logical given that reductions in fecundity for short-lived plants directly translate into a reduction in lifetime fitness, whereas the effects of herbivores on perennial plant fitness are more difficult to determine because perennials can compensate for herbivore damage across years [17]. Recruitment is crucial for the fitness of short-lived plants, as the majority of plant mortality occurs at this stage [54]. Our results on *M*. *moricandioides*, in which more than 90% of the individuals reproduce only once, support the evidence that herbivores can strongly influence the performance of short-lived plants.

Ungulates affected seed viability by decreasing seed mass in some arid and semiarid shrubs [23,25]. In our system, ungulates affected seed quality by reducing the content of carbon in the seeds, but there was no effect in seed mass or nitrogen content. This reduction in carbon content in seeds might explain the lower seed viability, and even the higher mortality of seedlings. Net carbon balance is necessary for plant growth and survival, and modest changes in carbon allocation patterns may have large consequences for seedling emergence and seedling survival [55]. Indeed, recent studies in *Arabidopsis* reveal that carbon-dependent signaling pathways could be ubiquitous regulators of seed germination and as important as nitrogen content and seed mass in determining germination success [56,57]. Carbon limitation in seeds could be a cost of resprouting after being grazed or trampled by ungulates, which immediately reduce the photosynthetic capacity and affect the rate of accumulation of water-soluble carbohydrates in the plant [58]. Ungulate damage does not only lead to tissue loss and thus losses of carbon, but also affects the stored reserves, which mostly consist of carbon resources [22]. Thus, ungulates limit the amount of carbon that could be allocated to seeds, because the resources used for regrowth may translate to fewer resources allocated to reproduction [59]. Our previous work also showed that floral herbivores alter carbon and nitrogen content in seeds of *M*. *moricandioides* resulting in a reduction in seedling emergence and establishment [40].

There was no prominent transgenerational effect of PSPs on *M*. *moricandioides*. In other systems, pre-dispersal seed predation reduces seedling emergence and recruitment in plant species differing in life cycle [37]. On the contrary, *M*. *moricandioides* plants attacked by PSPs overcompensated increasing seed production [Aguirrebengoa et al. under review, González-Megías et al. in prep.] Therefore, the lack of any transgenerational effect of PSPs on seeds support the idea that there is a positive net effect of PSPs on *M*. *moricandioides* and opens an interesting debate about how this presumably antagonistic interaction has evolved to become a "mutualistic" one. This type of interaction is even more difficult to explain in resource-limited environments where both the plant and the insects undergo extreme and unpredictable abiotic conditions.

### Effects of ungulates and seed predators on seedling herbivory and performance

Our study demonstrates significant transgenerational and interacting effects of ungulates and PSPs on plants by affecting the herbivory experienced by the offspring of the same host plant. Moreover, the transgenerational effects varied according to the herbivore feeding mode and their specialization degree, which suggests that multiple mechanisms are involved.

There are very few examples in the literature of maternal herbivory affecting the influences of other herbivore guilds on the offspring than the inducer herbivore (see [6]). We found that when the parental generation was affected by ungulates, the offspring was less susceptible to leaf miner attack. Host specificity and adaptations to plant defenses tend to be very high in this herbivore guild [60,61], and leaf miners are also quite sensitive to leaf nutrient content and quality [62–64]. Hence, the ungulate transgenerational effect on leaf miners may be the result of the lower carbon content in seeds that could be inherited in seedlings. Analyses of seedling leaf nutrient content and defenses will be necessary to elucidate the specific mechanism behind our results.

One of the most surprising results of our study is that PSPs and ungulates had an interacting transgenerational effect on *M*. *moricandioides* seedlings. When maternal plants suffered the pressure of both herbivores (ungulates and PSPs), seedlings increased resistance against generalist herbivores (grasshoppers). The fact that only generalist herbivores but not specialists ones (leaf miners and chrysomelids) were negatively affected by this interaction suggest that the combined impact of ungulate and PSP herbivory on maternal plants triggers a defense response in seedlings, because these are usually more effective against generalist than specialist herbivores [65,66]. Several studies show that maternal herbivory can induce physical (trichomes) and chemical defenses [9,11,13].

Transgenerational defense induction can depend on the degree of predictability of future attack [67]. According to [10], transgenerational induced resistance to herbivores could be expected in those plants subjected to a non-predictable attack rate (i.e. PSPs). On the contrary, if the herbivore attack rate remains constant over time (i.e. ungulates) a constitutive resistance could have evolved. Transgenerational defense induction to reduce herbivory on offspring would therefore be expected for PSPs but not so clearly for ungulates that remain constant for decades and mainly affect plants by trampling. Indeed, ungulate constant impact could have also favored the development of tolerance that allows survival and reproduction [22,68]. Other authors suggested that seedlings of stressed plants are able to rapidly induce defenses in response to a stress similar to the one suffered by the parental plants [69,70]. Seedlings have limited structures required for resource acquisition, and thus, they might rely more on induced rather than constitutive resistance [71,72]. By which features herbivores were deterred in offspring is difficult to elucidate and needs further studies.

### Biotic transgenerational effects on seeds and seedlings at within-plant level

Contrary to our prediction, we found no effect in seed traits and seedling performance of PSPs at within-plant level. Within-plant pre-dispersal seed predation effects have been observed in some trees [73,74]. A higher emergence rate of un-attacked fruit seeds due to nitrogen allocation to these fruits has been reported in *Mimosa bimucronata* [73]. Similarly, higher seed mass and emergence rate of un-attacked cone seeds have been observed in *Juniperus thurifera* [74]. All examples in the literature of within-plant variation on seed quality due to PSP pressure are from long-lived plants. Plant capacity for localized response within the plant may thus depend on life cycle and predictability of seed predator attack rates.

The most novel result arising from our study is that there was a within-plant transgenerational response to ungulates and PSPs. While some plant performance traits such as seedling emergence or survival were only affected at plant level, transgenerationally induced responses to herbivores occurred at both plant and within-plant levels, which may be related to within-plant variation in plant defense, i.e. glucosinolate induction in *M*. *moricandioides*. Defense induction in plants often occurs in the specific tissue damaged, and locally in the specific damaged part [66,75,76]. Regarding within-plant variation, a recent study shows that the patterns of DNA cytosine methylation in leaves are highly variable within individuals, and within-individual variance even surpasses the variance between individuals [77]. Through within-individual variation, plants probably better cope with the heterogeneous environment and optimize the exploitation of resources [39]. Therefore, within-plant variation in transcriptional responses may be caused by within-plant transgenerational differences in gene expression regulation or defence-inducing hormones [77].

## Conclusions

**Fig 3:**
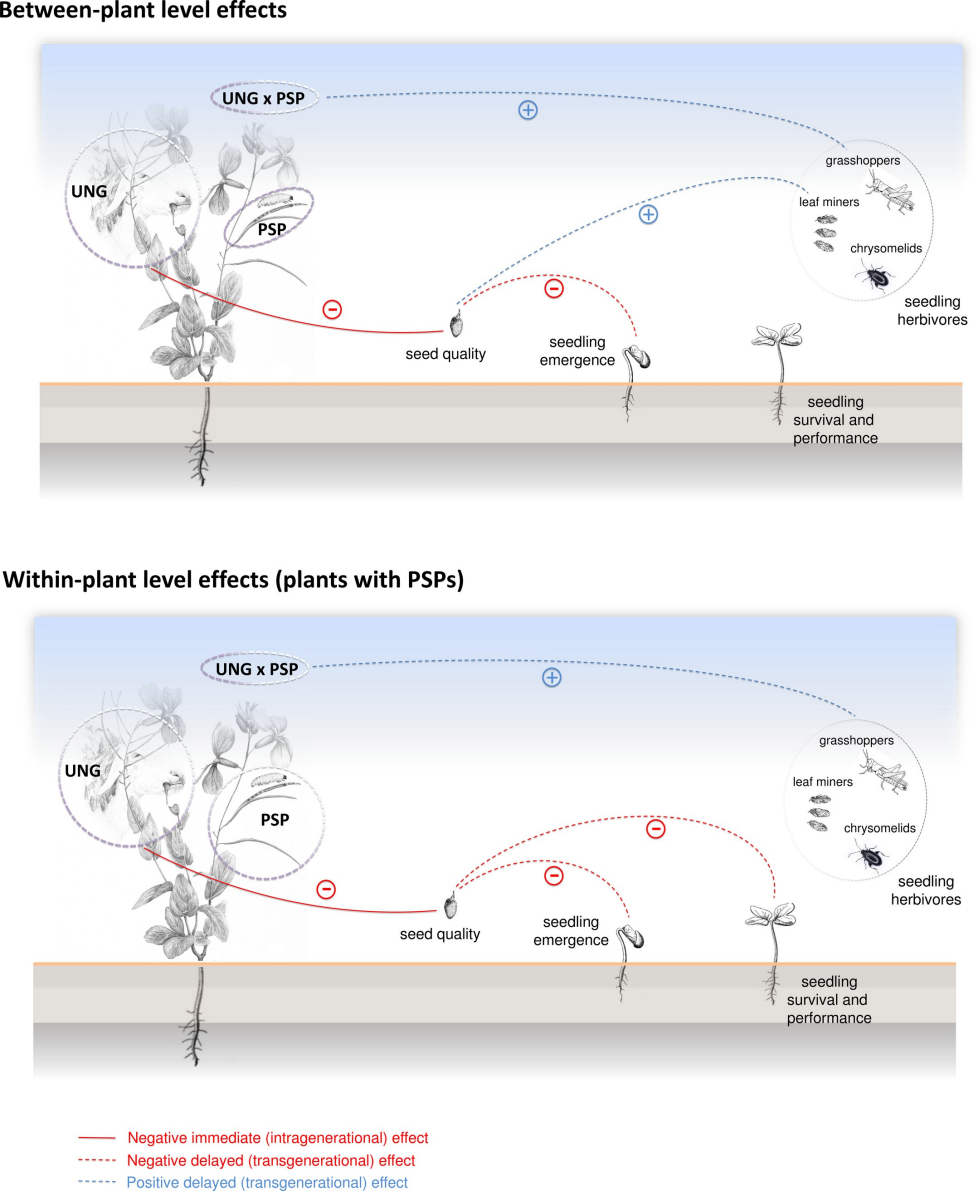
Illustrative figure of the observed transgenerational effects of ungulates and PSPs on *Moricandia moricandioides*. At between-plant level, we observed that ungulates reduced seed quality (carbon content) and seedling emergence rate, but these seedlings were more resistant to the attack by leaf miners. In addition, seedlings from mother plants exposed to ungulates and attacked by PSPs suffered less herbivory by grasshoppers. At within-plant level, the negative effect of ungulates was strengthened on plants with PSPs, as ungulates reduced seed quality and seedling emergence rate but also seedling survival rate. We observed variation in transgenerational-induced resistance among siblings, as seedlings from mother plants exposed to ungulates and from fruits attacked by PSPs were more resistant to herbivores than seedlings from the same plants from un-attacked fruits.

In summary, our results reinforce the idea that the interplay of biotic factors can be especially relevant on plant recruitment. Additionally, this study reveals the crucial importance of biotic maternal environment on the outcome of biotic interactions in resource-limited environments. Two herbivore types, very different in size and feeding strategy, could have independent but also interactive effects on seedling recruitment and herbivore damage, for which seed nutrient provisioning and transgenerational defense induction might be the main mechanisms (see Fig 3). This study is in line with other studies in which the complexity of ensemble effects of species interactions were found to be transgenerationally transmitted [40,78,79]. Finally, our results highlight that biotic transgenerational effect occurred not only at plant level but also at within-plant level, with siblings differing on their transgenerational-induced resistance to insect herbivory. This result underlines the need to consider biotic transgenerational effects and the intra-individual variability when studying the interaction between herbivores and plants.

## Supporting information

S1 Fig Experimental *Moricandia moricandioides* populations. Location map of the twelve experimental populations of *Moricandia moricandioides* in the study area (Barranco del Espartal, geographical coordinates 37° 31´ 12´´ N 2° 42´ 12´´ W). Blue points denote populations excluded from ungulates, red points denote populations exposed to ungulates. The map comes from the public ortophoto repository of the Department of the Environment of the Regional Government of Andalucía, Spain (Consejería de Medio Ambiente de la Junta de Andalucía, España). http://www.juntadeandalucia.es/medioambiente/site/rediam/menuitem.f361184aaadba3cf8ca78ca731525ea0/?vgnextoid=168a7c119370f210VgnVCM2000000624e50aRCRD&lr=lang_es.
(EPS)

S2 Fig Flow diagram of the study. Experimental design and sample sizes in each step of followed procedure: fruit collection, seed trait measurements, seedling emergence determination and field experiments with seedlings. Between-plant level effects refers to differences between (mother) plants, within-plant level effects refers to differences within each (mother) plant depending on whether they had PSPs in the fruits.
(TIF)

S1 Appendix Model selection at between-plant and within-plant levels. (PDF)
