# Supplementary figures and images for: Transgenerational effects of ungulates and pre-dispersal seed predators on offspring success and resistance to herbivory
Source: PLoS One. 2018 Dec 12;13(12):e0207553. doi: 10.1371/journal.pone.0207553 (PMC6291102; doi:10.1371/journal.pone.0207553)

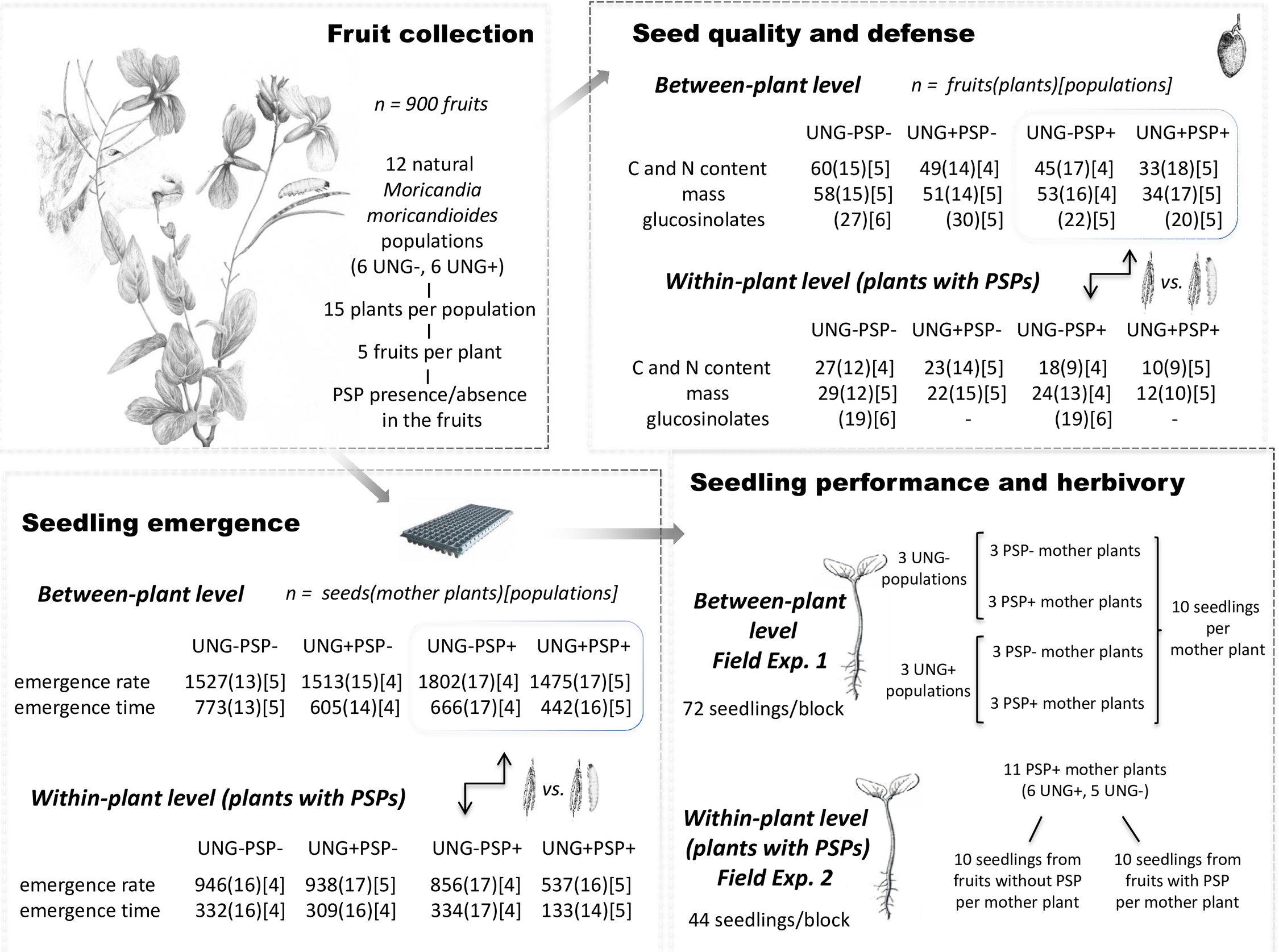

Supplement: S2 Fig — Experimental design and sample sizes in each step of followed procedure: fruit collection, seed trait measurements, seedling emergence determination and field experiments with seedlings. Between-plant level effects refers to differences between (mother) plants, within-plant level effects refers to differences within each (mother) plant depending on whether they had PSPs in the fruits. (TIF) [file pone.0207553.s002.tif]
