# Supplementary material for: Transgenerational effects of ungulates and pre-dispersal seed predators on offspring success and resistance to herbivory
Source: PLoS One. 2018 Dec 12;13(12):e0207553. doi: 10.1371/journal.pone.0207553 (PMC6291102; doi:10.1371/journal.pone.0207553)
Supplement: S1 Appendix — (PDF) [file pone.0207553.s003.pdf]

S1 Appendix. Model selection at between-plant and within-plant levels

Both maternal lineage (e.g. within-plant variation is not distributed equally among individuals [1], genetic variation in fitness impacts of herbivory [2], genetic variation in offspring responses to herbivory [3], genetic variation on epigenetic inducibility and/or phenotypic impact of epigenetic modifications [4]), and population origin (e.g. differences in the strength of abiotic and biotic selection [5], variation across populations in transgenerational phenotypic plasticity [6]) could influence the traits of interest. Thus, we tested several models with different random structures for seed and seedling variables with the aim to control origin effects.

Between-plant level models

	**Random structure**	**AIC**	**AIC weight**	**AICc**	**AICc weight**	**BIC**	**BIC weight**
**Seed mass**	Plant[Population] / Population[Ungulate]	-261.040	0.0259	-260.122	0.0211	-231.090	0.0003
	Plant / Population[Ungulate]	-263.040	0.0705	-262.309	0.0630	-236.417	0.0042
	Plant[Population]	-265.040	0.1915	-264.475	0.1861	-241.745	0.0609
	Plant / Population	-265.040	0.1915	-264.475	0.1861	-241.745	0.0609
	Plant	-267.040	0.5206	-266.618	0.5436	-247.073	0.8737

	**Random structure**	**AIC**	**AIC weight**	**AICc**	**AICc weight**	**BIC**	**BIC weight**
**Carbon in seeds**	Plant[Population] / Population[Ungulate]	840.428	0.0321	841.391	0.0261	869.977	0.0004
	Plant / Population[Ungulate]	838.428	0.0873	839.194	0.0782	864.694	0.0059
	Plant[Population]	837.060	0.1730	837.652	0.1690	860.042	0.0606
	Plant / Population	836.428	0.2373	837.021	0.2310	859.411	0.0830
	Plant	835.060	0.4703	835.502	0.4950	854.759	0.8501

	**Random structure**	**AIC**	**AIC weight**	**AICc**	**AICc weight**	**BIC**	**BIC weight**
**Nitrogen in seeds**	Plant[Population] / Population[Ungulate]	207.725	0.0838	208.687	0.0718	237.273	0.0033
	Plant / Population[Ungulate]	205.725	0.2279	206.491	0.2152	231.990	0.0467
	Plant[Population]	210.725	0.0185	211.340	0.0191	233.730	0.0196
	Plant / Population	203.725	0.6195	204.317	0.6381	226.707	0.6556
	Plant	208.746	0.0503	209.189	0.0558	228.446	0.2748

	Random structure	AIC	AIC weight	AICc	AICc weight	BIC	BIC weight
**C/N ratio in seeds**	Plant[Population] / Population[Ungulate]	629.088	0.0782	630.051	0.0666	658.637	0.0025
	Plant / Population[Ungulate]	627.088	0.2127	627.854	0.1997	653.354	0.0358
	Plant[Population]	630.686	0.0352	631.279	0.0360	653.668	0.0306
	Plant / Population	625.088	0.5782	625.681	0.5920	648.071	0.5021
	Plant	628.686	0.0957	629.128	0.1056	648.385	0.4290

	Random structure	AIC	AIC weight	AICc	AICc weight	BIC	BIC weight
**Aliphatic GLS in seeds**	Population[Ungulate]	-160.970	0.0000	-159.740	0	-142.805	0
	Population	-162.970	0.0000	-162.057	0	-147.400	0
		-183.064	0.9999	-182.419	1	-170.088	1

	Random structure	AIC	AIC weight	AICc	AICc weight	BIC	BIC weight
**Indolic GLS in seeds**	Population[Ungulate]	-263.826	0	-262.595	0	-245.660	0
	Population	-265.826	0	-264.913	0	-250.255	0
		-295.755	1	-295.110	1	-282.780	1

	Random structure	AIC	AIC weight	AICc	AICc weight	BIC	BIC weight
**Total GLS in seeds**	Population[Ungulate]	-161.871	0.0000	-160.640	0	-143.705	0
	Population	-163.871	0.0000	-162.958	0	-148.301	0
		-184.081	0.9999	-183.436	1	-171.106	1

	Random structure	AIC	AIC weight	AICc	AICc weight	BIC	BIC weight
**Emergence rate**	Mother plant[Population] / Population[Ungulate]	7020.923	0.0027	7020.946	0.0027	7074.931	0.0000
	Mother plant / Population[Ungulate]	7018.923	0.0074	7018.941	0.0074	7066.180	0.0005
	Mother plant[Population]	7033.025	0.0000	7033.039	0.0000	7073.531	0.0000
	Mother plant / Population	7016.923	0.0201	7016.936	0.0202	7057.429	0.0412
	Mother plant	7031.025	0.0000	7031.035	0.0000	7064.780	0.0010
	Mother plant[Population] / Population[Ungulate] / Planting day	7016.035	0.0314	7016.064	0.0312	7076.794	0.0000
	Mother plant / Population[Ungulate] / Planting day	7014.035	0.0853	7014.058	0.0850	7068.043	0.0002
	Mother plant[Population] / Planting day	7012.690	0.1671	7012.708	0.1670	7059.947	0.0117
	Mother plant / Population / Planting day	7012.035	0.2318	7012.053	0.2316	7059.292	0.0162
	Mother plant / Planting day	7010.690	0.4542	7010.703	0.4549	7051.196	0.9291

	Random structure	AIC	AIC weight	AICc	AICc weight	BIC	BIC weight
**Emergence time**	Mother plant[Population] / Population[Ungulate]	15256.04	0.0292	15256.10	0.0289	15302.60	0.0001
	Mother plant / Population[Ungulate]	15254.04	0.0794	15254.09	0.0792	15294.78	0.0033
	Mother plant[Population]	15257.40	0.0148	15257.43	0.0149	15292.32	0.0114
	Mother plant / Population	15252.04	0.2158	15252.08	0.2164	15286.96	0.1667
	Mother plant	15255.40	0.0403	15255.43	0.0406	15284.50	0.5709
	Mother plant[Population] / Population[Ungulate] / Planting day	15257.00	0.0181	15257.08	0.0178	15309.38	0.0000
	Mother plant / Population[Ungulate] / Planting day	15255.00	0.0491	15255.06	0.0487	15301.56	0.0001
	Mother plant[Population] / Planting day	15253.34	0.1129	15253.39	0.1126	15294.07	0.0048
	Mother plant / Population / Planting day	15253.00	0.1335	15253.05	0.1332	15293.74	0.0056
	Mother plant / Planting day	15251.34	0.3069	15251.37	0.3078	15286.26	0.2370

	Random structure	AIC	AICc	BIC
**Field Exp. 1 – Survival rate**	Mother plant / Block	318.719	318.957	342.019
	Mother plant	316.820	316.990	336.237

	Random structure	AIC	AICc	BIC
**Field Exp. 1 – Produced leaves**	Mother plant / Block	1172.423	1172.742	1199.606
	Mother plant	1170.423	1170.661	1193.722

	Distribution	Random structure	AIC	AICc	BIC
**Field Exp. 1 – Leaves attacked by chrysomelids**		Mother plant / Block	438.482	438.801	465.665
	Zero-inflated Poisson	Mother plant	436.482	436.720	459.781

	Distribution	Random structure	AIC	AICc	BIC
**Field Exp. 1 – Leaves attacked by leaf miners**	Zero-inflated Poisson	Mother plant / Block	261.166	261.855	288.242
		Mother plant	259.168	259.701	282.859

	Distribution	Random structure	AIC	AICc	BIC
**Field Exp. 1 – Leaves attacked by grasshoppers**	Zero-inflated Negative Binomial	Mother plant / Block	1007.254	1007.665	1038.321
		Mother plant	1007.446	1007.765	1034.629

	Distribution	Random structure	AIC	AICc	BIC
**Field Exp. 1 – Leaves attacked in total**	Zero-truncated Negative Binomial	Mother plant / Block	-2804.06	-2803.741	-2776.877
		Mother plant	-2293.90	-2293.661	-2270.877

Within- plant level models (Random structure: PSP presence/absence nested to maternal plant identity)

	Additional random structure	AIC	AIC weight	AICc	AICc weight	BIC	BIC weight
**Seed mass**	Plant[Population] / Population[Ungulate]	-101.061	0.0259	-97.955	0.0140	-72.739	0.0009
	Plant / Population[Ungulate]	-103.061	0.0705	-100.503	0.0502	-77.314	0.0085
	Plant[Population]	-105.061	0.1915	-102.992	0.1741	-81.889	0.0836
	Plant / Population	-105.061	0.1915	-102.992	0.1741	-81.889	0.0836
	Plant	-107.061	0.5206	-105.425	0.5876	-86.463	0.8234

	Additional random structure	AIC	AIC weight	AICc	AICc weight	BIC	BIC weight
**Carbon in seeds**	Plant[Population] / Population[Ungulate]	371.338	0.0649	374.812	0.0368	398.589	0.0041
	Plant / Population[Ungulate]	369.338	0.1763	372.195	0.1360	394.111	0.0388
	Plant[Population]	371.043	0.0752	373.351	0.0763	393.339	0.0571
	Plant / Population	367.338	0.4793	369.646	0.4866	389.634	0.3641
	Plant	369.043	0.2044	370.866	0.2644	388.862	0.5358

	Additional random structure	AIC	AIC weight	AICc	AICc weight	BIC	BIC weight
**Nitrogen in seeds**	Plant[Population] / Population[Ungulate]	133.222	0.0260	136.695	0.0130	160.472	0.0010
	Plant / Population[Ungulate]	131.222	0.0707	134.079	0.0482	155.995	0.0093
	Plant[Population]	129.229	0.1913	131.537	0.1717	151.525	0.0869
	Plant / Population	129.222	0.1921	131.529	0.1724	151.518	0.0873
	Plant	127.229	0.5200	129.052	0.5947	147.048	0.8155

	Additional random structure	AIC	AIC weight	AICc	AICc weight	BIC	BIC weight
**C/N ratio in seeds**	Plant[Population] / Population[Ungulate]	314.001	0.0265	317.475	0.0133	341.252	0.0010
	Plant / Population[Ungulate]	312.001	0.0721	314.858	0.0492	336.775	0.0095
	Plant[Population]	310.066	0.1897	312.374	0.1705	332.362	0.0867
	Plant / Population	310.001	0.1960	312.309	0.1762	332.297	0.0895
	Plant	308.066	0.5157	309.889	0.5908	327.885	0.8132

	Additional random structure	AIC	AICc	BIC
**Aliphatic GLS in seeds**	Plant[Population]	-52.750	-50.875	-44.562
	Plant	-54.750	-53.537	-48.199

	Additional random structure	AIC	AICc	BIC
**Indolic GLS in seeds**	Plant[Population]	-152.941	-151.066	-144.753
	Plant	-154.941	-153.729	-148.391

	Additional random structure	AIC	AICc	BIC
**Total GLS in seeds**	Plant[Population]	-52.939	-51.064	-44.751
	Plant	-54.939	-53.727	-48.389

	Additional random structure	AIC	AIC weight	AICc	AICc weight	BIC	BIC weight
**Emergence rate**	Mother plant[Population] / Population[Ungulate]	3510.000	0.0006	3510.067	0.0006	3570.947	0.0000
	Mother plant / Population[Ungulate]	3508.000	0.0017	3508.055	0.0017	3562.852	0.0002
	Mother plant[Population]	3513.912	0.0001	3513.956	0.0001	3562.669	0.0002
	Mother plant / Population	3506.000	0.0048	3506.044	0.0048	3554.758	0.0087
	Mother plant	3511.912	0.0002	3511.946	0.0002	3554.574	0.0096
	Mother plant[Population] / Population[Ungulate] / Planting day	3502.624	0.0257	3502.705	0.0253	3569.665	0.0000
	Mother plant / Population[Ungulate] / Planting day	3500.624	0.0699	3500.691	0.0694	3561.571	0.0003
	Mother plant[Population] / Planting day	3498.624	0.1901	3498.679	0.1897	3553.476	0.0166
	Mother plant / Population / Planting day	3498.624	0.1901	3498.679	0.1897	3553.476	0.0166
	Mother plant / Planting day	3496.624	0.5167	3496.668	0.5185	3545.381	0.9480

	Additional random structure	AIC	AIC weight	AICc	AICc weight	BIC	BIC weight
**Emergence time**	Mother plant[Population] / Population[Ungulate]	6801.210	0.0036	6801.374	0.0036	6846.303	0.0002
	Mother plant / Population[Ungulate]	6798.755	0.0123	6798.919	0.0122	6843.848	0.0008
	Mother plant[Population]	6797.534	0.0227	6797.665	0.0228	6837.616	0.0175
	Mother plant / Population	6796.755	0.0334	6796.886	0.0337	6836.838	0.0259
	Mother plant	6795.534	0.0616	6795.636	0.0630	6830.606	0.5833
	Mother plant[Population] / Population[Ungulate] / Planting day	6798.020	0.0178	6798.221	0.0173	6848.124	0.0001
	Mother plant / Population[Ungulate] / Planting day	6795.540	0.0614	6795.740	0.0597	6845.643	0.0003
	Mother plant[Population] / Planting day	6793.540	0.1669	6793.704	0.1654	6838.633	0.0105
	Mother plant / Population / Planting day	6793.540	0.1669	6793.704	0.1654	6838.633	0.0105
	Mother plant / Planting day	6791.540	0.4536	6791.671	0.4570	6831.622	0.3509

	Additional random structure	AIC	AICc	BIC
**Field Exp. 2 – Survival rate**	Mother plant / Block	304.150	304.839	331.226
	Mother plant	303.714	304.247	327.405

	Additional random structure	AIC	AICc	BIC
**Field Exp. 2 – Produced leaves**	Mother plant / Block	833.751	834.616	864.211
	Mother plant	835.084	835.773	862.160

	Distribution	Additional random structure	AIC	AICc	BIC
**Field Exp. 2 – Leaves attacked by chrysomelids**		Mother plant / Block	232.182	233.966	276.180
	Zero-inflated Poisson	Mother plant	242.256	242.945	269.332

	Distribution	Additional random structure	AIC	AICc	BIC
**Field Exp. 2 – Leaves attacked by leaf miners**	Zero-inflated Poisson	Mother plant / Block	262.104	262.969	292.564
		Mother plant	260.102	260.791	287.178

	Distribution	Additional random structure	AIC	AICc	BIC
**Field Exp. 2 – Leaves attacked by grasshoppers**	Zero-inflated Negative Binomial	Mother plant / Block	658.120	658.985	688.580
		Mother plant	673.446	673.979	697.137

	Distribution	Additional random structure	AIC	AICc	BIC
**Field Exp. 2 – Leaves attacked in total**	Zero-truncated Negative Binomial	Mother plant / Block	-1304.512	-1303.978	-1280.821
		Mother plant	-973.036	-972.637	-952.729

S1 Appendix References

1. Herrera CM. The ecology of subindividual variability in plants: patterns, processes, and prospects. Web Ecol. 2017;17(2):51–64.

2. Strauss AA, Agrawal S. The ecology and evolution of plant tolerance to herbivory. Trends Ecol Evol. 1999;14(5):179–85.

3. Agrawal AA. Herbivory and maternal effects: Mechanisms and consequences of transgenerational induced plant resistance. Ecology. 2002;83(12):3408–15.

4. Holeski LM, Zinkgraf MS, Couture JJ, Whitham TG, Lindroth RL. Transgenerational effects of herbivory in a group of long-lived tree species: maternal damage reduces offspring allocation to resistance traits, but not growth. J Ecol. 2013;101(4):1062–73.

5. Colautti RI, Lee C-R, Mitchell-Olds T. Origin, fate, and architecture of ecologically relevant genetic variation. Curr Opin Plant Biol. 2012; 15(2):199–204.

6. Colicchio J. Transgenerational effects alter plant defence and resistance in nature. J Evol Biol. 2017;30(4):664–80.
